# Prediction of subnational-level vaccination coverage estimates using routine surveillance data and survey data

**DOI:** 10.1016/j.vaccine.2025.127277

**Published:** 2025-07-11

**Authors:** Deepit Bhatia, Natasha Crowcroft, Sébastien Antoni, M. Carolina Danovaro-Holliday, Anindya Sekhar Bose, Anna Minta, Balcha Masresha, Matthew J. Ferrari

**Affiliations:** a Pennsylvania State University, United States; b WHO-IVB, Switzerland; c WHO-AFR, Republic of Congo

**Keywords:** Measles, Vaccination coverage estimation, Beta regression, Out-of-sample prediction, DHS

## Abstract

**Background:**

Measles vaccination has significantly reduced the global burden of the disease, but disparities in vaccination coverage persist. Accurate and timely estimates of subnational vaccination coverage are crucial for identifying high-risk areas and guiding targeted interventions. However, existing methods face limitations related to accuracy, timeliness, and spatial resolution. We explored the use of routinely collected case-based surveillance data to predict measles vaccination coverage at the subnational level.

**Methods:**

The study used aggregated case data from 18 countries in the WHO African region, obtained from the WHO measles surveillance database. Three surveillance-based indicators were derived: mean age of suspected measles cases, proportion of vaccinated suspected cases, and proportion of IgM-negative suspected cases. These indicators were used to build a beta regression model with measles vaccination coverage from the Demographic and Health Surveys (DHS) as the gold standard. We compared out-of-sample predictions created using this model to withheld DHS estimates using Pearson's rho.

**Findings:**

We found that each of the three surveillance-based indicators were more strongly correlated with DHS-based survey coverage than administrative estimates. Out-of-sample predictions achieved high correlation with DHS-based coverage, with a rho of 0.74.

**Interpretation:**

The findings suggest that routinely collected measles surveillance data can effectively predict subnational measles vaccination coverage. The approach addresses limitations of existing methods by providing yearly estimates that are more accurate than administrative data and more readily available than surveys. This enables timely identification of low-coverage areas and facilitates targeted interventions.

## Highlights

• Inequities persist in vaccination coverage against measles.
• Identifying areas of low coverage is crucial in preventing future outbreaks.
• Existing measures have a trade-off between accuracy and timeliness.
• We estimate subnational coverage based on characteristics of suspected cases.
• Estimates are well correlated with survey-based measures and can be generated yearly.

## 1 Introduction

Immunization with measles-containing vaccines, through routine immunization and supplemental campaigns, has led to a reduced burden of measles, averting 57 million deaths from 2000 to 2022 [1]. However, reductions in the burden of measles have not been equal across the world, with the highest incidence and lowest routine immunization coverage observed in the World Health Organization (WHO) African Region and further differences in coverage both between and within countries [2,3]. As coverage has increased in this region, the rate of increase has slowed, and rapidly identifying areas with low coverage is necessary to prevent outbreaks and increase equity [4].

Within-country variation in routine vaccination coverage and the performance of supplemental campaigns can affect the persistence of transmission and the overall coverage levels necessary to meet or exceed the critical herd immunity threshold [5]. Quantifying such heterogeneity can be useful in identifying priority areas for outbreak monitoring and response and targeting of preventive strategies to supplement routine vaccination activities [6].

Currently available methods for assessing subnational vaccination coverage reflect inherent trade-offs between accuracy and timeliness (or expense). Administrative vaccination coverage is calculated as the number of doses administered to a specific age group in a specific area divided by the estimated number of people living in that age group in that area [7,8]. While administrative coverage is frequently available at the subnational level at regular (often yearly) intervals, its accuracy can be limited, though efforts are underway to improve the quality of administrative indicators [9,10]. Administrative coverage can result in biased estimates of the proportion vaccinated by 1) counting immunizations outside the target population and 2) failing to account for immunizations administered in private clinics [7]. Critically, estimates of the target population could also be inaccurate or out of date [6,7]; thus, even when doses administered are counted accurately, the estimate of coverage can be biased by an inaccurate denominator.

Survey-based coverage estimates, on the other hand, are derived from a sample of visited households in a specific area, and information is collected (via recall or through existing documentation) on the vaccine status of children in specific age groups [7]. Estimates from large and rigorously conducted probability-based surveys (e.g. Demographic and Health Surveys (DHS), the Multi-Cluster Indicator Survey (MICS), and EPI cluster survey [[11], [12], [13]] can be more accurate and provide formal estimates of uncertainty [14]. While survey-based measures are considered accurate and have high spatial resolution, they are not timely, with surveys conducted roughly every 3–5 years if at all [11]. The large time intervals between surveys mean that estimates of coverage for countries within any given supranational region are rarely coincident and estimates from across multiple years must be combined to assess sub-national coverage [15]. Survey-based methods are also subject to biases separate from administrative data, such as inaccurate home-based records, recall bias, inflated coverage measures due to shared inaccessibility in administering vaccines and surveys, and increasing imprecision at higher spatial resolutions for a given overall sample size [7,14].

Composite estimates have been employed to combine administrative and survey-based data and address some of the limitations of each individual source. The WUENIC (WHO/UNICEF estimates of national immunization coverage) estimates calibrate national administrative data using survey-based estimates when and where they exist [16]; at present, these estimates are available for all countries every year, but, unlike administrative or survey-based measures, are not available at the subnational level. The Institute for Health Metrics and Evaluation (IHME) Local Burden of Disease project uses a statistical model to predict coverage at a 5kmx5km resolution using surveys and national and sub-national level covariates [17]. While this allows subnational estimation of past coverage at different times, it is dependent on covariates that may not be publicly available or up-to-date for creating future estimates [17]. Furthermore, few validation exercises have been conducted [18].

Given the tradeoffs evident in existing coverage measures, we explore whether routinely collected case-based surveillance data on suspected measles cases could be used to predict measles vaccine coverage at the subnational level. Case-based surveillance data are not currently being utilized to estimate coverage but contain several attributes that have a theoretical link to the transmission dynamics of measles. Thus, even if the functional form of the relationship between these surveillance-based indicators and coverage is not known, we expect three attributes in particular: mean age of suspected cases, vaccine coverage among suspected cases with known vaccination status, and negative IgM test results among suspected cases, to be positively correlated with vaccine coverage [[19], [20], [21], [22]]. Furthermore, as these three attributes are summary measures of reported cases, we anticipate that they should be robust to uniform underreporting of cases.

### 1.1 Mean age (of suspected measles cases with known age)

In the absence of vaccination, the mean age of infection is inversely proportional to R_0_ [19]. The mean age of infection is expected to increase as vaccination coverage increases in successive birth cohorts, because susceptible individuals are less likely to encounter infection during any year of their life when the effective reproductive number, R_E_, is lower [19,20]. This can be described mathematically as:

$$A=\frac{L}{\left(1-C*\mathrm{VE}\right)R_{0}-1}$$

where *A* is the mean age of infection and L is the life expectancy, *C* is vaccination coverage, and *VE* is vaccine efficacy.

### 1.2 Vaccination coverage (among suspected cases with known vaccination status)

As vaccination coverage increases, a higher fraction of cases occurring will be among individuals who failed to generate a protective immune response (primary failure) [22]. In addition, as vaccination coverage increases a higher fraction of suspected cases will be due to non-measles sources of febrile rash (e.g., rubella virus) among previously measles vaccinated individuals.

### 1.3 Negative IgM test results (among tested suspected cases)

The proportion of suspected cases that are diagnostically negative for measles (i.e., IgM negative) is expected to increase as measles vaccination coverage increases. Assuming that other sources of febrile rash (e.g., rubella virus, parvovirus, dengue fever, and varicella zoster virus) that meet the clinical case definition remain in the population, these sources should increase in relative proportion to diagnostically confirmed measles cases as vaccination increases and measles prevalence decreases [21].

Existing measures of coverage are limited in spatial resolution, temporal resolution, availability, or accuracy. Here we propose coverage estimates based on surveillance-based indicators that are routinely collected and available at a high spatial resolution within months of collection. The resulting of sub-national measles vaccination coverage may prove useful in the absence of regularly occurring vaccination coverage surveys.

## 2 Methods

### 2.1 Data

We used aggregated case data from 18 countries in the WHO African region to derive our covariates of interest and used aggregated survey measles vaccination data from the DHS as our outcome of interest. We limited our analyses to those countries in the WHO African region where DHS data were also available for at least two iterations between 2007 and 2019 to allow for out-of-sample validation. These countries are Benin, Burundi, Cameroon, Democratic Republic of Congo, Ethiopia, Ghana, Guinea, Kenya, Lesotho, Liberia, Malawi, Mozambique, Nigeria, Rwanda, Sierra Leone, Tanzania, Zambia, and Zimbabwe.

Routine measles surveillance data are submitted by Member States to the WHO on an ongoing, provisional basis. Member States report individual-level data about suspected measles cases seeking healthcare, and include characteristics such as age, sex, prior vaccination history, and immunoglobulin M (IgM) test results. Suspected measles cases are defined as those with a fever and a maculopapular (non-vesicular) rash and at least one of cough, coryza, or conjunctivitis [23]. Data for this analysis were obtained from the WHO measles surveillance database on 2 May 2021 and 2 November 2021 through a request to the Immunizations, Vaccines, and Biologicals (IVB) Department. For each year, country, and first-level administrative ADM1 unit WHO IVB provided counts of 1) the total number of cases meeting the suspected measles case definition by each year of age, 2) the number of suspected cases that reported a prior measles containing vaccine dose, 3) the number of suspected cases IgM positive for measles, and 4) the number of suspected cases tested for measles IgM. To remove possible coding errors in age, we removed all entries where the reported age was 90 years or older. Furthermore, ADM1 regions that did not have at least 30 cases reported in the WHO case surveillance data in any rolling three-year period were excluded. From 1) we calculated the mean age of suspected measles cases per 3 year rolling period (e.g., 2007–2009, 2008–2010, 2009–2011, etc.) for each ADM1 unit. From 1) and 2) we calculated the proportion of suspected measles cases in each period and ADM1 unit that reported at least 1 prior measles containing vaccination. From 3) and 4) we calculated the proportion of IgM tested cases that were negative for measles IgM antibodies in each period and ADM1 unit.

We used ADM1 level data on childhood measles vaccination from the DHS [11]. The surveys used spanned the years 2007–2019, with the year of the survey corresponding to the last year in a 3-year period of WHO surveillance data described above. The list of surveys included and the years they were conducted is presented in Supplementary Table 1. We used the percentage of children aged 12–23 months of age who received at least 1 dose of the measles containing vaccine (MCV) at any time in the past, according to a documented vaccination card or to the child's caregiver as outcome of interest [11]. We estimated the mean and standard error of coverage, on the logit scale, from the DHS survey results for each ADM1 unit by building intercept-only quasibinomial logistic regression models as described in [25]. Additionally, we used subnational-level administrative data as provided by member states to the WHO in our comparisons of the validity of our methodology.

In both the DHS and case-surveillance data sources, we aggregated spatial units into larger units when necessary to ensure that data would be comparable across time. (see Supplementary Table 5 for a detailed description). We then calculated the Pearson correlation coefficient for each of the three surveillance-based and DHS coverage using R statistical software [24].

### 2.2 In-sample and out-of-sample prediction of coverage

We built a random intercept beta regression model with the *glmmTMB* package in R, using DHS-derived coverage as the outcome, and the 3 surveillance-based indicators as the predictors [24]. Beta regression was used because coverage is restricted to the open interval *[0,1]*, as we did not expect any ADM-1 regions in our study region had a true coverage of exactly 0 % or 100 %. The intercept for each country was treated as a random effect to account for country-level differences. We generated predicted coverage for all ADM-1 units in these 19 countries for 2018–2020 by applying *predict()* in package *glmmTMB* using the corresponding surveillance-based indicators as co-variates using all available covariate data.

To assess out-of-sample predictions, we re-fit the above model using outcomes and corresponding covariates only for the year of the first DHS iteration for each country. To account for the uncertainty due to sampling in the DHS outcome data we then refit the full regression model using 100 bootstrapped draws of the DHS ADM1 coverage, where each draw was an inverse logit transformation of a sample from a normal distribution with mean and standard error derived from the intercept-only models described above. We then used *predict()* in package *glmmTMB* to predict the coverage in the year of the second DHS survey for each country using the surveillance-based indicators for the corresponding year as covariates for each of the 100 bootstrap-derived model coefficients. We then compared the predicted, out-of-sample coverage values with DHS-derived coverage values using Pearson's correlation coefficient and the root mean square error (RMSE).

## 3 Results

Within the analysis dataset, spanning at least 2 DHS surveys for each included country, a total of **376,682 reported cases** were observed. To predict coverage, all three surveillance measures were required for each observation corresponding to an ADM1 unit and a year where a DHS survey took place. This information was available for 396 of 417 (95.0 %) of ADM1 units, encompassing **376,624 of 376,682, or 99.98 %** of all suspected cases. We calculate mean age from all suspected cases with a record of age (*N* = 361,532) and note that this is highly correlated (Pearson's rho = 0.71) with the mean age of IgM confirmed measles cases (*N* = 34,382; Supplementary Fig. 1). The analysis below is presented in terms of the mean age of suspected cases as that is the more readily available data in the absence of consistent laboratory confirmation. Within the DHS data, information was available for 416 of 417 observations, where data from the “Sud Ouest” region of Cameroon from the 2018 DHS Survey was unavailable due to data quality concerns [11].

Subnational-level administrative coverage was poorly correlated with survey-based coverage (Pearson's rho of 0.16) from 2016 to 2020 in the study region. Of all ADM1 region-years with both measures of coverage available, 26 of the 116 (22.4 %) ADM1 region-years had administrative coverage values of over 100 %, while no region-years had administrative coverage values of <50 %. Of the ADM1 units with reported admin coverage >100, the range of DHS coverage is 40.5 % to 97.8 % (IQR:60 % to 91.4 %), suggesting that administrative coverage estimates of >100 % do not necessarily imply high coverage.

**Fig. 1:**
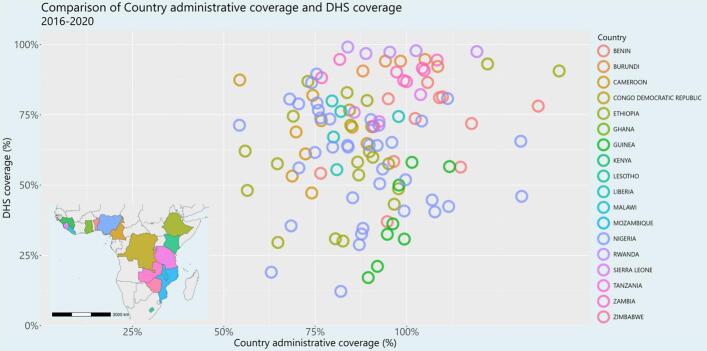
Comparison of subnational-level administrative coverage (x-axis) and survey-based MCV1 coverage (y-axis) from 2016 to 2020. **Map production:** World Health Organization, 2024. All rights reserved. **Data Source:** IVB Database. **Disclaimer:** The boundaries and names shown and the designations used on this map do not imply the expression of any opinion whatsoever on the part of any of the authors or the institutions they are affiliated with concerning the legal status of any country, territory, city, or area or its authorities, or concerning the delimitation of its frontiers and boundaries. Dotted and dashed lines on maps represent approximate border lines for which there may not yet be full agreement.

**Fig. 2:**
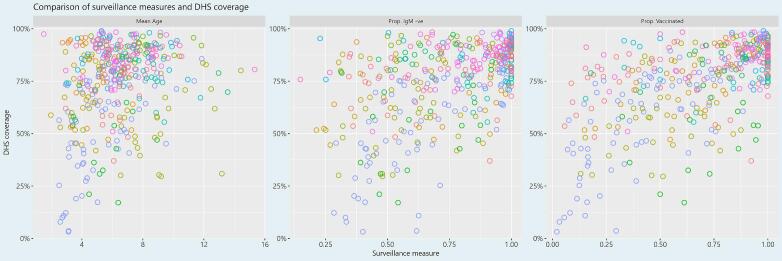
Comparison of subnational-level surveillance indicators (left to right: mean age, proportion IgM negative, and proportion vaccinated) and survey-based coverage. See Fig. 1 for the legend.

The three subnational-level surveillance measures of mean age, proportion IgM negative, and proportion vaccinated among suspected measles cases with known vaccination status were better correlated with survey-based MCV1 coverage (Pearson's rho of 0.24 [95 %CI: 0.14, 0.33], 0.50 [95 %CI: 0.42, 0.57], 0.61 [95 %CI: 0.54, 0.67] respectively) compared to the correlation between administrative and survey-based coverage (See Fig. 2). Restricting this comparison only to the years for which there are both DHS survey estimates and administrative coverage estimates (as in Fig. 1), this correlation still holds (Pearson's rho of 0.19 [95 %CI: 0.01, 0.37], 0.71 [95 %CI: 0.61, 0.79], and 0.68 [95 %CI: 0.56, 0.77] respectively).

**Fig. 3:**
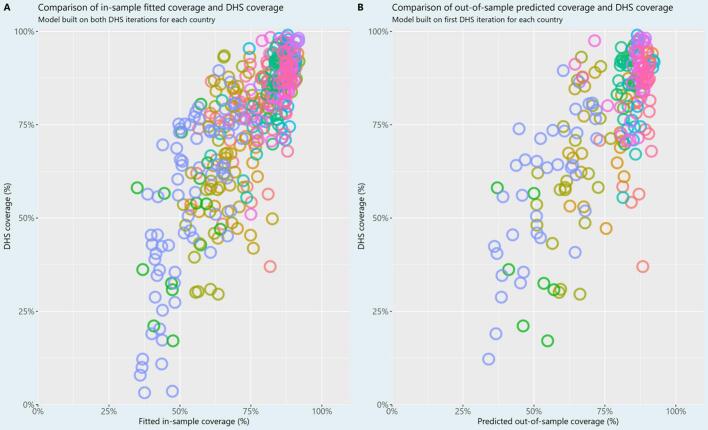
Comparison of vaccination coverage obtained through fitting all observations (L), and coverage predicted out-of-sample (R) with survey-based coverage. See Fig. 1 for the legend.

Vaccine coverage values derived from the regression model fitted on all data were well-correlated (Pearson's rho of 0.76, 95 % CI = 0.79 to 0.83) with survey-based MCV1 coverage. When we fit the model only to the first DHS iteration in our dataset and predict out-of-sample coverage values for the subsequent DHS iterations, the correlation remains high, with Pearson's rho of 0.74 (95 % CI = 0.68 to 0.82) (see Fig. 3). Among 100 model runs sampling from the distribution of initial DHS outcome values, 95 % of values were between 0.726 and 0.752. The out-of-sample prediction RMSE was 0.131, with 95 % of values between 0.128 and 0.134 (see Supplementary Figs. 2, 3). Mean age and proportion previously vaccinated were consistently statistically significant at the 0.05 level in our model. While proportion IgM negative was not statistically significant at the 0.05 level in the model with all three covariates, it was a statistically significant covariate in models without proportion previously vaccinated, due to the high degree of correlation between the two covariates (see Supplementary Table 1).

Coverage values predicted using the model trained on the first DHS iteration range from less than 40 % to greater than 90 % in 2018 to 2020. Notably, coverage remains consistently high within Benin, Ghana, Rwanda, Tanzania, and Zimbabwe across this period. Lower coverage estimates are predicted for DRC and Ethiopia, with a notable degree of heterogeneity between administrative units. Predicted coverage varies most dramatically in Nigeria, with estimates ranging from 31 % in Borno to 89 % in Ekiti.

## 4 Discussion

**Fig. 4:**
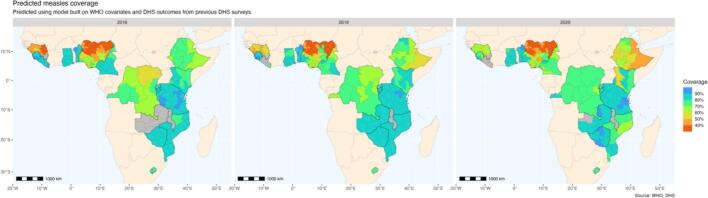
Out-of-sample predicted coverage in the study area in 2018 (left), 2019 (middle), and 2020 (right). **Map production:** World Health Organization, 2024. All rights reserved. **Data Source:** IVB Database. **Disclaimer:** The boundaries and names shown and the designations used on this map do not imply the expression of any opinion whatsoever on the part of any of the authors or the institutions they are affiliated with concerning the legal status of any country, territory, city, or area or its authorities, or concerning the delimitation of its frontiers and boundaries. Dotted and dashed lines on maps represent approximate border lines for which there may not yet be full agreement.

Using routinely collected measles surveillance data backed by a simple regression model, we predicted yearly MCV1 vaccination coverage at a subnational level. Predicted estimates created using our model address the trade-offs present in currently available coverage measures; our estimates are both more accurate than other yearly estimates of coverage, and are more frequently calculable than accurate, probability-based cluster survey measures of coverage. Importantly, using surveillance data enables predictions of coverage for years where no health surveys were done (see Fig. 4).

Unlike national-level estimates of administrative coverage, publicly available subnational-level estimates are not triangulated with survey-based estimates. As a result, 22.4 % of subnational estimates between 2016 and 2020 were reported to be >100 %. Unadjusted subnational-level administrative coverage estimates have the potential to significantly underestimate risk by overestimating coverage and can be misleading for risk assessments [8].

Of the three subnational-level measles surveillance measures (mean age, proportion IgM negative, and proportion vaccinated), mean age was the measure with the highest availability (96.0 %), but had the poorest correlation with DHS coverage. This may be due to patients with other diseases with similar symptoms to measles, (e.g., rubella), seeking care and being recorded as a suspected measles case (note: of the included countries, Cameroon, Ghana, Kenya, Mozambique, Rwanda, Tanzania, Zambia, and Zimbabwe have used rubella containing vaccine (RCV) as of 2018). Proportion IgM negative and vaccination status were available for a smaller proportion of cases (29.5 % and 61.7 % respectively). Due to low availability of IgM status, we used the mean age of all suspected cases in our model (Supplementary Table 2).

Restricting mean age only to IgM negative cases would not necessarily yield less biased estimates, as any testing strategies focusing on specific ages would bias the mean age covariate. Increased availability of rapid diagnostic tests will also allow for more complete data and increase the potential to use the mean age of only IgM-confirmed cases, which would increase the predictive power of the mean age covariate in the model.

While the availability of prior vaccination status is currently low, further standardization of how this question is recorded at the point of care presents an opportunity to rapidly improve completeness and the quality and timeliness of coverage estimates in the future. Currently, vaccination status is recorded via maternal recall or vaccination card/home-based record; recall is prone to underreporting and low specificity to vaccine type [26], and vaccination card suffers quality issues, issues in availability, low retention, and retention associated with sociodemographic factors including child age, child gender, and household wealth [27]. Improvements in recording vaccination status, e.g. the implementation of electronic registries, could alleviate some of the biases in recall- or card-based methods of reporting coverage and also allow mitigating gaps in coverage at the local level due to richer individual-level data [28]. However, most low and middle-income countries are far from being able to implement such registries [29].

A limitation in our methodology is that coverage predicted through our fitted model does not fall below ∼30 %, even when survey-based coverage is observed to be below 30 %. This may be due to the small sample of countries that included in this study—a larger number of countries could allow for better estimation of the random intercept term, potentially allowing for predicted values outside of the currently observed lower bounds. Also, country-level coverage measures are not as consistently well-correlated with predictions. This may be because countries with a high overall level of coverage have low variability in coverage between administrative subunits (i.e., high coverage overall, and in each administrative subunit). Another limitation in our methodology is that acute changes in measles epidemiology or vaccine delivery may result in rapid changes in coverage and possibly delayed responses in the surveillance indicators which may change the predictive ability of the model in ways we are not yet able to predict. Furthermore, due to the lack of temporally concomitant survey data for each country, we were unable to account for explicit temporal and spatial correlation in the analyses and addressed the variation in within- and between-country differences by using a country-level random intercept in our model.

Fitting our model requires that the country have a high-quality benchmark of coverage at least one time point to estimate the random intercept. Between 2007 and 2023, the DHS has been conducted in 72 countries [30]. Furthermore, surveillance-based estimates rely on health data to be collected and reported by healthcare facilities. We assume that suspected cases seeking care at healthcare facilities have similar reported as the overall population. By using summary characteristics of cases that were reported, we expect the impact of poor data collection and reporting to be minimized, compared to using incidence as a covariate in estimating coverage.

Our methodology complements existing methods of estimating coverage, by using a data source not commonly used in existing measures–routine surveillance data. Other estimates of coverage such as the IHME Local Burden of Disease estimates, derive information from survey-based measures, supplemented by covariates related to social determinants of health. By incorporating covariates, they can also be used in areas where direct data collection is not feasible, for example due to conflict [31]. While covariate-based measures have produced results at a high spatial resolution, their value in predicting coverage to monitor progress towards different targets may be limited, due to the possibility of one or more of the many data sources used in creating the estimates being unavailable in the future, or due to the timescale required in planning, implementing, analyzing, and publishing survey-based estimates upon which these estimates rely [18].

With correlation observed between survey coverage and surveillance-based indicators, it is possible that surveillance-based indicators could be better monitored to increase data completeness as they are used to provide specific insights on different sources of vaccination, and on the ability of health systems to adequately vaccinate children against vaccine-preventable diseases generally. We might expect short-term changes in the mean age, proportion IgM positive, or proportion previously vaccinated among suspected cases as a result of supplemental immunization activities (SIAs). While it is possible that these indicators may change slowly, *Minetti et. al* showed lower relative risk of infection among age groups targeted by an outbreak response vaccination campaign [32]. More broadly, measles is considered a “tracer”. Due to the highly infectious nature of measles, outbreaks occur rapidly in areas with low vaccination coverage [1]. Thus, post-campaign analysis of surveillance data, or enhanced surveillance after a campaign could be a useful addition to campaign evaluation.

We assume that cases that are seen by healthcare facilities are roughly representative of the cases experienced in the communities overall in terms of mean age of cases, proportion of cases with known vaccination history vaccinated against measles, and proportion of cases with confirmatory negative IgM test results). We would obtain less precise measures of coverage using our methods if, in the community, the mean age, proportion vaccinated, or proportion of individuals meeting the case definition but without measles infection, was consistently different from what was observed (and recorded) in the healthcare setting. Despite potential biases in the surveillance indicators due to access and reporting, they remain useful in predicting coverage.

## 5 Conclusion

Routinely collected disease surveillance data can be used to estimate vaccine coverage with a combination of accuracy and timeliness not available in existing methods. Our methodology is computationally simple, relies only on data collected routinely in national public health surveillance systems, and has the potential to improve in its predictive power with increased data collection and the advent of cheaper point-of-care rapid diagnostic tests. In sum, the method described here with three elements of surveillance data: age, MCV vaccination status and IgM results, which are generally readily available and continuously updated, provide the national or sub-national level program manager another tool to identify areas with low measles coverage and implement appropriate response measures and evaluate progress.

## CRediT authorship contribution statement

**Deepit Bhatia:** Writing – review & editing, Writing – original draft, Visualization, Project administration, Methodology, Formal analysis, Data curation, Conceptualization. **Natasha Crowcroft:** Writing – review & editing, Supervision, Conceptualization. **Sébastien Antoni:** Writing – review & editing, Resources, Project administration, Investigation, Data curation. **M. Carolina Danovaro-Holliday:** Writing – review & editing, Resources, Investigation. **Anindya Sekhar Bose:** Writing – review & editing, Resources, Project administration, Investigation. **Anna Minta:** Writing – review & editing, Resources, Investigation. **Balcha Masresha:** Writing – review & editing, Resources, Investigation. **Matthew J. Ferrari:** Writing – review & editing, Writing – original draft, Supervision, Resources, Project administration, Methodology, Funding acquisition, Conceptualization.

## Disclaimer

The authors alone are responsible for the views expressed in this article and they do not necessarily represent the views, decisions or policies of the institutions with which they are affiliated.

## Funding

This work was supported, in whole or in part, by the Bill & Melinda Gates Foundation Investment number 016091. Under the grant conditions of the Foundation, a Creative Commons Attribution 4.0 Generic License has already been assigned to the Author Accepted Manuscript version that might arise from this submission. DB and MF were also funded by NSF-NIH-NIFA Ecology and Evolution of Infectious Disease award DEB 1911962.

## Declaration of competing interest

The authors declare that they have no known competing financial interests or personal relationships that could have appeared to influence the work reported in this paper.

## Data Availability

Proposals for aggregated case-based covariates should be directed to the WHO to gain access, data requestors will need to sign a data access agreement.

## Appendix A Supplementary data

Supplementary material Image 1
