# Supplementary material for: Prediction of subnational-level vaccination coverage estimates using routine surveillance data and survey data
Source: Vaccine. 2025 Jul 11;60:None. doi: 10.1016/j.vaccine.2025.127277 (PMC12161133; doi:10.1016/j.vaccine.2025.127277)
Supplement: Supplementary file 1 — Supplementary material [file mmc1.docx]

# Title

Prediction of subnational-level vaccination coverage estimates using routine surveillance data and survey data

# Supplement

## Supplementary Methods

### Beta regression:

Beta regression models assume that the outcome data $Y$ follow a Beta distribution with a mean $\mu$, variance $V$, and a precision parameter $\phi$ shown below.

$$Y \sim Beta(\mu,\phi)$$

$$V\left[ Y \right]= \frac{\mu(1- \mu)}{1+ \phi}$$

The regression equation is as follows, where $i$ represents measures from a specific year and subnational subunit, $j$ represents country, and 1, 2, and 3 represent mean age of suspected cases, proportion testing negative among suspected cases IgM tested for measles, and the proportion vaccination among suspected cases with known vaccination history respectively.

$$g\left( \mu\right)=\beta_{j}+ \beta_{1}X_{1(i,j)}+ \beta_{2}X_{2(i,j)}+ \beta_{3}X_{3(i,j)}$$

Using the logit link function $g\left( \mu\right) = ln \left( \frac{\mu}{1-\mu} \right)$. Using the inverse logit function $g^{-1}\left( x \right) = \frac{e^{x}}{1+e^{x}}$. , we can arrive at the mean $\mu$ as:

$$g^{-1}\left( g\left( \mu\right) \right)=\mu$$

$$g^{-1}\left( \beta_{j}+ \beta_{1}X+ \beta_{1}X_{2}+ \beta_{1}X_{3} \right)=\mu$$

Model call in R:

b_oos_repeat3<- glmmTMB(dhs_coverage ~ p_vax + meanAge + p_igm_neg + (1|country_name),

test_dataset, family=beta_family())

## Supplementary results

|  | **DHS Coverage** | **Mean Age** | **Proportion Vaccinated** | **Proportion IgM Negative** | **Admin Coverage** |
| --- | --- | --- | --- | --- | --- |
| **DHS Coverage** |  | 0.240* | 0.610* | 0.500* | 0.160 |
| **Mean Age** |  |  | 0.160* | 0.100 | -0.020 |
| **Proportion Vaccinated** |  |  |  | 0.690 | 0.190 |
| **Proportion IgM Negative** |  |  |  |  | 0.083 |

Supplementary Table 1: Pearson’s correlation coefficients between coverage estimates and case characteristics. Highly significant (p < 0.005) correlations are marked with *.


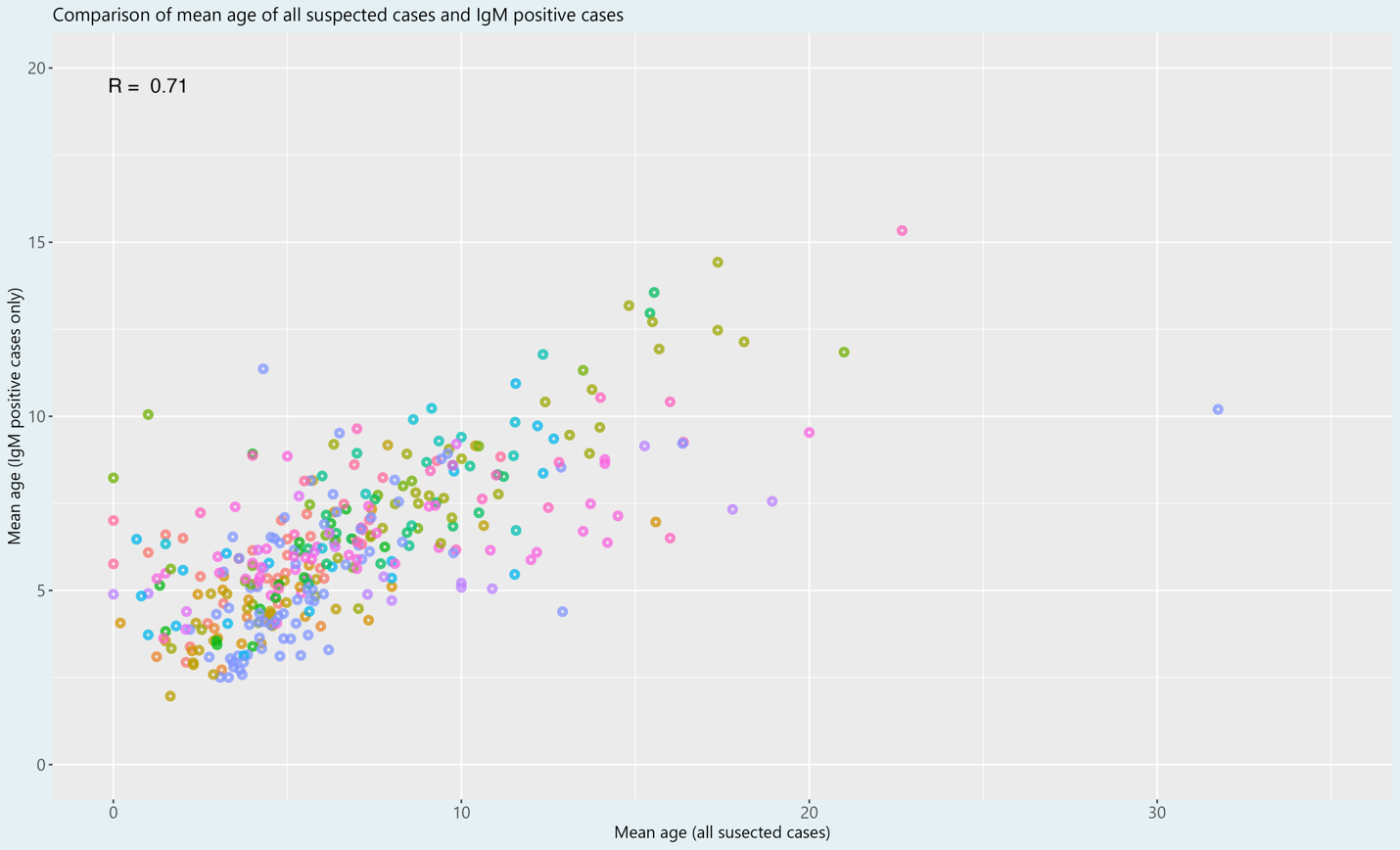


Supplementary Figure 1: Correlation between mean age of all suspected cases and mean age of IgM positive cases.


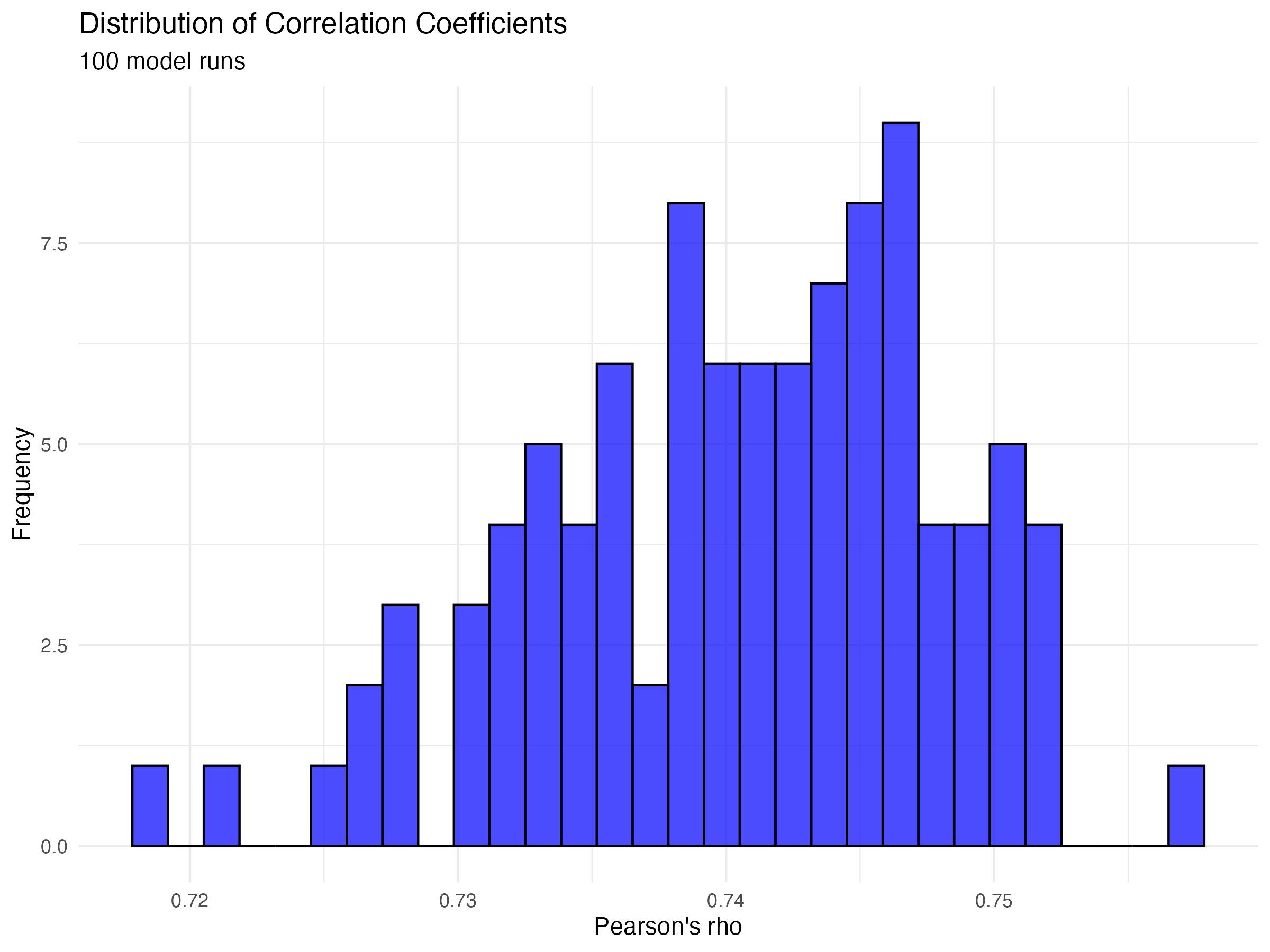


Supplementary Figure 2: Distribution of Pearson's correlation coefficient between the predicted ADM1 coverage and the observed ADM1 coveage for the out-of-sample survey in each country. Each value reflects the results of 1 of 100 model fits to random draws from the distribution of ADM1 coverage from the first DHS survey for each country, used to predict the coverage value in the second survey for the corresponding ADM1 unit.


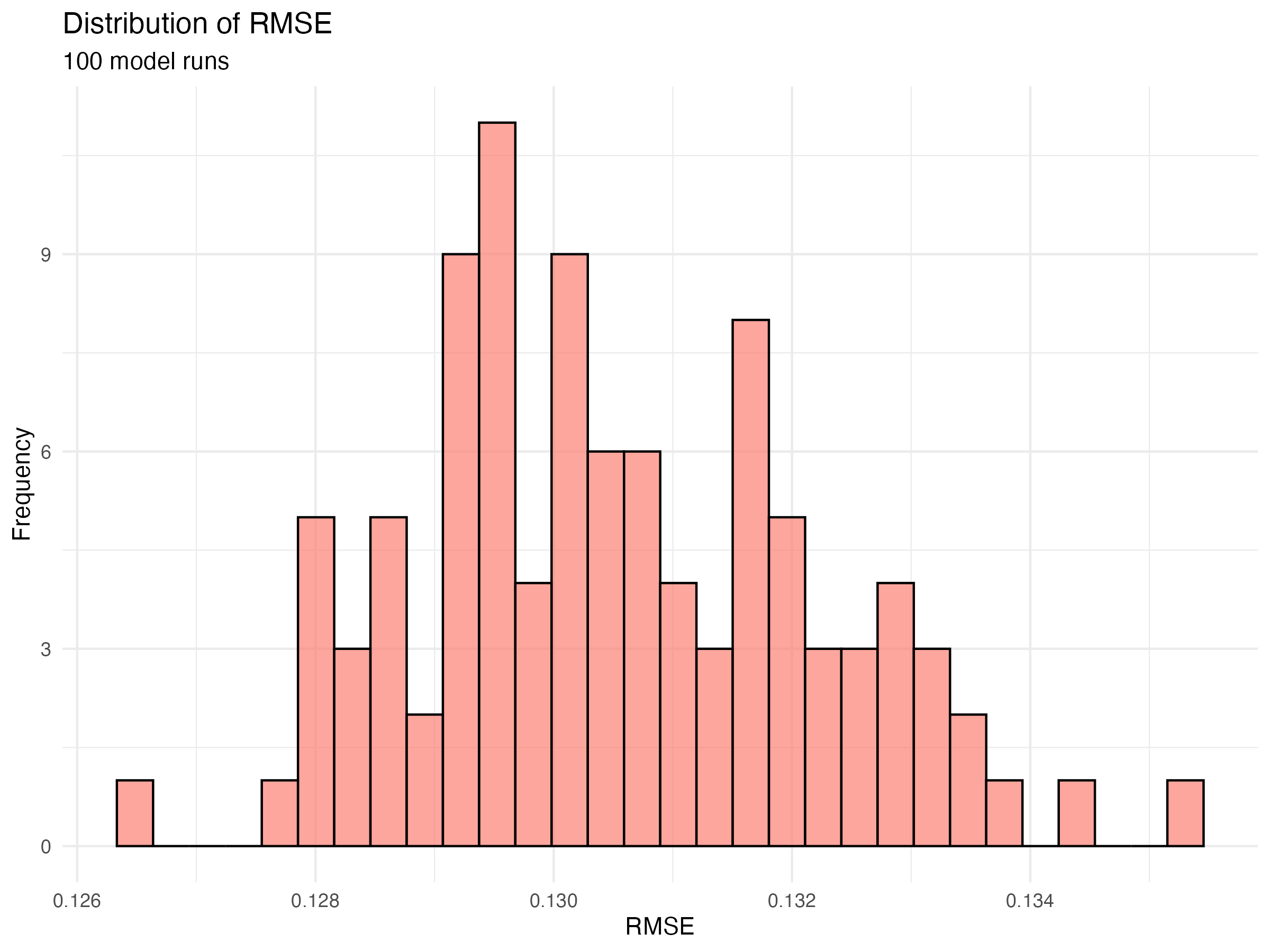


Supplementary Figure 3: Distribution of root mean square error (RMSE) between the predicted ADM1 coverage and the observed ADM1 coveage for the out-of-sample survey in each country. Each value reflects the results of 1 of 100 model fits to random draws from the distribution of ADM1 coverage from the first DHS survey for each country, used to predict the coverage value in the second survey for the corresponding ADM1 unit.

| **Country** | **ADM-1 units** | **DHS years** | **Cases** | **Cases with age** | **Cases with vaccination status** | **Cases IgM tested** | **% cases with age** | **% cases with vaccination status** | **% cases IgM tested** | **Total ADM1-years** | **ADM1-years missing age** | **ADM1-years missing vaccination status** | **ADM1-years without IgM testing** | **ADM-1 years without missing age, vaccination status, or IgM testing** |
| --- | --- | --- | --- | --- | --- | --- | --- | --- | --- | --- | --- | --- | --- | --- |
| BENIN | 12 | 2012, 2017 | 2,506 | 2,503 | 1,441 | 1,880 | 99.9% | 57.5% | 75.0% | 24 | 0 | 0 | 0 | 24 |
| BURUNDI | 5 | 2010, 2016 | 1,432 | 1,430 | 1,352 | 902 | 99.9% | 94.4% | 63.0% | 10 | 0 | 0 | 0 | 10 |
| CAMEROON | 10 | 2011, 2018 | 8,621 | 8,617 | 7,115 | 5,187 | 100.0% | 82.5% | 60.2% | 20 | 0 | 0 | 0 | 20 |
| CONGO DEMOCRATIC REPUBLIC | 11 | 2007, 2013 | 14,695 | 13,778 | 7,972 | 8,908 | 93.8% | 54.2% | 60.6% | 22 | 0 | 0 | 0 | 22 |
| ETHIOPIA | 11 | 2011, 2016, 2019 | 73,146 | 72,105 | 46,809 | 25,731 | 98.6% | 64.0% | 35.2% | 33 | 0 | 0 | 0 | 33 |
| GHANA | 10 | 2008, 2014 | 4,863 | 4,017 | 2,533 | 3,639 | 82.6% | 52.1% | 74.8% | 20 | 0 | 0 | 0 | 20 |
| GUINEA | 8 | 2012, 2018 | 2,833 | 2,794 | 1,385 | 2,318 | 98.6% | 48.9% | 81.8% | 16 | 1 | 1 | 1 | 15 |
| KENYA | 8 | 2008, 2014 | 12,459 | 11,456 | 8,883 | 6,500 | 91.9% | 71.3% | 52.2% | 16 | 0 | 0 | 0 | 16 |
| LESOTHO | 10 | 2009, 2014 | 1,607 | 1,605 | 930 | 810 | 99.9% | 57.9% | 50.4% | 20 | 0 | 0 | 0 | 20 |
| LIBERIA | 5 | 2013, 2019 | 3,321 | 3,316 | 828 | 704 | 99.8% | 24.9% | 21.2% | 10 | 1 | 1 | 1 | 9 |
| MALAWI | 3 | 2010, 2015 | 76,244 | 75,763 | 1,746 | 2,692 | 99.4% | 2.3% | 3.5% | 6 | 0 | 0 | 0 | 6 |
| MOZAMBIQUE | 11 | 2011, 2015 | 7,106 | 7,036 | 2,353 | 4,757 | 99.0% | 33.1% | 66.9% | 22 | 0 | 0 | 0 | 22 |
| NIGERIA | 37 | 2013, 2018 | 139,558 | 130,545 | 129,774 | 34,150 | 93.5% | 93.0% | 24.5% | 74 | 0 | 0 | 0 | 74 |
| RWANDA | 5 | 2008, 2010, 2015, 2019 | 4,012 | 3,988 | 3,399 | 3,694 | 99.4% | 84.7% | 92.1% | 20 | 8 | 8 | 8 | 12 |
| SIERRA LEONE | 4 | 2013, 2019 | 1,044 | 1,028 | 690 | 585 | 98.5% | 66.1% | 56.0% | 8 | 0 | 0 | 0 | 8 |
| TANZANIA | 26 | 2010, 2015 | 6,344 | 6,122 | 3,973 | 3,319 | 96.5% | 62.6% | 52.3% | 52 | 6 | 6 | 7 | 45 |
| ZAMBIA | 8 | 2007, 2013, 2018 | 4,388 | 4,039 | 2,258 | 1,632 | 92.0% | 51.5% | 37.2% | 24 | 3 | 4 | 3 | 20 |
| ZIMBABWE | 10 | 2010, 2015 | 12,503 | 11,390 | 8,972 | 3,564 | 91.1% | 71.8% | 28.5% | 20 | 0 | 0 | 0 | 20 |
| **Total** | **194** | **Total** | **376,682** | **361,532** | **232,413** | **110,972** | **96.0%** | **61.7%** | **29.5%** | **417** | **19** | **20** | **20** | **396** |

Supplementary Table 2: Data completeness for each country.

| **Country** | **DHS years** | **ADM-1 units** | **Notes** |
| --- | --- | --- | --- |
| BENIN | 2012, 2017 | 12 |  |
| BURUNDI | 2010, 2016 | 5 |  |
| CAMEROON | 2011, 2018 | 10 | DHS data for the Sud Ouest region was unavailable for 2018 (National Institute of Statistics (Cameroon) & ICF, 2020). |
| CONGO DEMOCRATIC REPUBLIC | 2007, 2013 | 11 |  |
| ETHIOPIA | 2011, 2016, 2019 | 11 |  |
| GHANA | 2008, 2014 | 10 |  |
| GUINEA | 2012, 2018 | 8 |  |
| KENYA | 2008, 2014 | 8 |  |
| LESOTHO | 2009, 2014 | 10 |  |
| LIBERIA | 2013, 2019 | 5 | The 2013 survey was used as the first DHS iteration, and 2019 as the second due to low case counts in surveillance data corresponding to the 2007 survey, and low data completeness in surveillance indicators corresponding to the 2013 survey. |
| MALAWI | 2010, 2015 | 3 |  |
| MOZAMBIQUE | 2011, 2015 | 11 |  |
| NIGERIA | 2013, 2018 | 37 |  |
| RWANDA | 2008, 2010, 2015, 2019 | 5 |  |
| SIERRA LEONE | 2013, 2019 | 4 | The 2013 survey was used as the first DHS iteration due to low case counts in surveillance data corresponding to the 2007 survey. |
| TANZANIA | 2010, 2015 | 26 |  |
| ZAMBIA | 2007, 2013, 2018 | 8 |  |
| ZIMBABWE | 2010, 2015 | 10 |  |
| Total | Total | 194 |  |

Supplementary Table 3: Details of DHS iterations used.

| **Country** | **Correlation** | **p** | **95%CI (lower)** | **95%CI (upper)** | **National-level coverage in out-of-sample data (%)** |
| --- | --- | --- | --- | --- | --- |
| BENIN | -0.028 | 0.931 | -0.592 | 0.555 | 67.9 |
| BURUNDI | -0.180 | 0.770 | -0.917 | 0.834 | 93.8 |
| CAMEROON | 0.670 | 0.046 | 0.019 | 0.924 | 65.3 |
| CONGO DEMOCRATIC REPUBLIC | 0.490 | 0.127 | -0.157 | 0.842 | 71.6 |
| ETHIOPIA | 0.510 | 0.015 | 0.113 | 0.767 | 54.3, 58.5 |
| GHANA | 0.068 | 0.852 | -0.587 | 0.669 | 89.3 |
| GUINEA | -0.520 | 0.232 | -0.915 | 0.383 | 61.8 |
| KENYA | 0.490 | 0.215 | -0.325 | 0.889 | 87.1 |
| LESOTHO | -0.030 | 0.936 | -0.647 | 0.611 | 90.1 |
| LIBERIA | 0.490 | 0.406 | -0.694 | 0.958 | 73.8 |
| MALAWI | 0.420 | 0.724 |  |  | 91.3 |
| MOZAMBIQUE | 0.310 | 0.352 | -0.355 | 0.768 | 82.7 |
| NIGERIA | 0.720 | 0.000 | 0.523 | 0.849 | 54.0 |
| RWANDA | 0.800 | 0.010 | 0.289 | 0.956 | 95.0, 95.2, 97.8 |
| SIERRA LEONE | 0.820 | 0.177 | -0.661 | 0.996 | 74.7 |
| TANZANIA | 0.140 | 0.518 | -0.287 | 0.524 | 86.0 |
| ZAMBIA | -0.110 | 0.727 | -0.645 | 0.493 | 90.9 |
| ZIMBABWE | -0.570 | 0.087 | -0.882 | 0.097 | 81.9 |

Supplementary Table 4: Country-level Pearson’s correlation between observed DHS coverage estimates, and predicted out-of-sample coverage estimates.

| **Aggregation Used** | **WHO Country** | **DHS Country** | **WHO Name** | **DHS Name** |
| --- | --- | --- | --- | --- |
| **alibori** | BENIN | Benin | ALIBORI | ..Alibori |
| **atacora** | BENIN | Benin | ATACORA | ..Atacora |
| **atlantique** | BENIN | Benin | ATLANTIQUE | ..Atlantique |
| **borgou** | BENIN | Benin | BORGOU | ..Borgou |
| **collines** | BENIN | Benin | COLLINES | ..Collines |
| **couffo** | BENIN | Benin | COUFFO | ..Couffo |
| **donga** | BENIN | Benin | DONGA | ..Donga |
| **littoral** | BENIN | Benin | LITTORAL | ..Littoral (Cotonou) |
| **mono** | BENIN | Benin | MONO | ..Mono |
| **oueme** | BENIN | Benin | OUEME | ..Ouémé |
| **plateau** | BENIN | Benin | PLATEAU | ..Plateau |
| **zou** | BENIN | Benin | ZOU | ..Zou |
| **west** | BURUNDI | Burundi | BUBANZA | West |
| **bujumbura mairie** | BURUNDI | Burundi | BUJUMBURA MAIRIE | Bujumbura Mairie |
| **west** | BURUNDI | Burundi | BUJUMBURA RURAL (21769-2) | West |
| **south** | BURUNDI | Burundi | BURURI (21769-1) | South |
| **centre-east** | BURUNDI | Burundi | CANKUZO | Centre-East |
| **west** | BURUNDI | Burundi | CIBITOKE | West |
| **centre-east** | BURUNDI | Burundi | GITEGA | Centre-East |
| **centre-east** | BURUNDI | Burundi | KARUSI | Centre-East |
| **north** | BURUNDI | Burundi | KAYANZA | North |
| **north** | BURUNDI | Burundi | KIRUNDO | North |
| **south** | BURUNDI | Burundi | MAKAMBA | South |
| **centre-east** | BURUNDI | Burundi | MURAMVYA | Centre-East |
| **north** | BURUNDI | Burundi | MUYINGA | North |
| **south** | BURUNDI | Burundi | MWARO | South |
| **north** | BURUNDI | Burundi | NGOZI | North |
| **south** | BURUNDI | Burundi | RUMONGE | South |
| **south** | BURUNDI | Burundi | RUTANA | South |
| **centre-east** | BURUNDI | Burundi | RUYIGI | Centre-East |
| **adamaoua** | CAMEROON | Cameroon | ADAMAOUA (21769-1) | ..Adamaoua |
| **centre** | CAMEROON | Cameroon | CENTRE (21769-1) | ..Centre |
| **centre** | CAMEROON | Cameroon | CENTRE (21769-1) | ..Yaoundé |
| **est** | CAMEROON | Cameroon | EST (21769-1) | ..Est |
| **extreme nord** | CAMEROON | Cameroon | EXTREME NORD (21769-2) | ..Extrême-Nord |
| **littoral** | CAMEROON | Cameroon | LITTORAL (21769-1) | ..Douala |
| **littoral** | CAMEROON | Cameroon | LITTORAL (21769-1) | ..Littoral |
| **nord** | CAMEROON | Cameroon | NORD (21769-1) | ..Nord |
| **nord ouest** | CAMEROON | Cameroon | NORD OUEST (21769-2) | ..Nord Ouest |
| **ouest** | CAMEROON | Cameroon | OUEST (21769-2) | ..Ouest |
| **sud** | CAMEROON | Cameroon | SUD (21769-1) | ..Sud |
| **sud ouest** | CAMEROON | Cameroon | SUD OUEST (21769-1) | ..Sud Ouest |
| **bandundu** | DEMOCRATIC REPUBLIC OF THE CONGO | Congo Democratic Republic | BANDUNDU | Bandundu |
| **orientale** | DEMOCRATIC REPUBLIC OF THE CONGO | Congo Democratic Republic | BAS UELE | Orientale |
| **bas-congo** | DEMOCRATIC REPUBLIC OF THE CONGO | Congo Democratic Republic | BAS-CONGO | Bas-Congo |
| **equateur** | DEMOCRATIC REPUBLIC OF THE CONGO | Congo Democratic Republic | EQUATEUR | Equateur |
| **katanga** | DEMOCRATIC REPUBLIC OF THE CONGO | Congo Democratic Republic | HAUT KATANGA | Katanga |
| **katanga** | DEMOCRATIC REPUBLIC OF THE CONGO | Congo Democratic Republic | HAUT LOMAMI | Katanga |
| **orientale** | DEMOCRATIC REPUBLIC OF THE CONGO | Congo Democratic Republic | HAUT UELE | Orientale |
| **orientale** | DEMOCRATIC REPUBLIC OF THE CONGO | Congo Democratic Republic | ITURI | Orientale |
| **kasai-occidental** | DEMOCRATIC REPUBLIC OF THE CONGO | Congo Democratic Republic | KASAI | Kasaï Occident |
| **kasai-occidental** | DEMOCRATIC REPUBLIC OF THE CONGO | Congo Democratic Republic | KASAI CENTRAL | Kasaï Occident |
| **kasai-oriental** | DEMOCRATIC REPUBLIC OF THE CONGO | Congo Democratic Republic | KASAI ORIENTAL | Kasaï Oriental |
| **kasai-occidental** | DEMOCRATIC REPUBLIC OF THE CONGO | Congo Democratic Republic | KASAI-OCCIDENTAL | Kasaï Occident |
| **kasai-oriental** | DEMOCRATIC REPUBLIC OF THE CONGO | Congo Democratic Republic | KASAI-ORIENTAL | Kasaï Oriental |
| **katanga** | DEMOCRATIC REPUBLIC OF THE CONGO | Congo Democratic Republic | KATANGA | Katanga |
| **kinshasa** | DEMOCRATIC REPUBLIC OF THE CONGO | Congo Democratic Republic | KINSHASA | Kinshasa |
| **bas-congo** | DEMOCRATIC REPUBLIC OF THE CONGO | Congo Democratic Republic | KONGO CENTRAL | Bas-Congo |
| **bandundu** | DEMOCRATIC REPUBLIC OF THE CONGO | Congo Democratic Republic | KWANGO | Bandundu |
| **bandundu** | DEMOCRATIC REPUBLIC OF THE CONGO | Congo Democratic Republic | KWILU | Bandundu |
| **kasai-oriental** | DEMOCRATIC REPUBLIC OF THE CONGO | Congo Democratic Republic | LOMAMI | Kasaï Oriental |
| **katanga** | DEMOCRATIC REPUBLIC OF THE CONGO | Congo Democratic Republic | LUALABA | Katanga |
| **bandundu** | DEMOCRATIC REPUBLIC OF THE CONGO | Congo Democratic Republic | MAINDOMBE | Bandundu |
| **maniema** | DEMOCRATIC REPUBLIC OF THE CONGO | Congo Democratic Republic | MANIEMA | Maniema |
| **equateur** | DEMOCRATIC REPUBLIC OF THE CONGO | Congo Democratic Republic | MONGALA | Equateur |
| **nord-kivu** | DEMOCRATIC REPUBLIC OF THE CONGO | Congo Democratic Republic | NORD KIVU | Nord-Kivu |
| **equateur** | DEMOCRATIC REPUBLIC OF THE CONGO | Congo Democratic Republic | NORD UBANGI | Equateur |
| **nord-kivu** | DEMOCRATIC REPUBLIC OF THE CONGO | Congo Democratic Republic | NORD-KIVU | Nord-Kivu |
| **orientale** | DEMOCRATIC REPUBLIC OF THE CONGO | Congo Democratic Republic | ORIENTALE | Orientale |
| **kasai-oriental** | DEMOCRATIC REPUBLIC OF THE CONGO | Congo Democratic Republic | SANKURU | Kasaï Oriental |
| **equateur** | DEMOCRATIC REPUBLIC OF THE CONGO | Congo Democratic Republic | SUD UBANGI | Equateur |
| **sud-kivu** | DEMOCRATIC REPUBLIC OF THE CONGO | Congo Democratic Republic | SUD-KIVU | Sud-Kivu |
| **katanga** | DEMOCRATIC REPUBLIC OF THE CONGO | Congo Democratic Republic | TANGANYIKA | Katanga |
| **orientale** | DEMOCRATIC REPUBLIC OF THE CONGO | Congo Democratic Republic | TSHOPO | Orientale |
| **equateur** | DEMOCRATIC REPUBLIC OF THE CONGO | Congo Democratic Republic | TSHUAPA | Equateur |
| **addis ababa** | ETHIOPIA | Ethiopia | ADDIS ABABA (21769-2) | Addis Ababa |
| **afar** | ETHIOPIA | Ethiopia | AFAR (21769-2) | Afar |
| **amhara** | ETHIOPIA | Ethiopia | AMHARA (21769-2) | Amhara |
| **benishangul-gumuz** | ETHIOPIA | Ethiopia | BENISHANGUL GUMU | Benishangul-Gumuz |
| **dire dawa** | ETHIOPIA | Ethiopia | DIRE DAWA (21769-1) | Dire Dawa |
| **gambela** | ETHIOPIA | Ethiopia | GAMBELLA (21769-1) | Gambela |
| **harari** | ETHIOPIA | Ethiopia | HARARI (21769-2) | Harari |
| **oromia** | ETHIOPIA | Ethiopia | OROMIA | Oromia |
| **oromia** | ETHIOPIA | Ethiopia | OROMIYA | Oromia |
| **snnpr** | ETHIOPIA | Ethiopia | SNNPR | SNNPR |
| **somali** | ETHIOPIA | Ethiopia | SOMALI (21769-1) | Somali |
| **tigray** | ETHIOPIA | Ethiopia | TIGRAY (21769-2) | Tigray |
| **brong-ahafo** | GHANA | Ghana | AHAFO | Brong-Ahafo |
| **ashanti** | GHANA | Ghana | ASHANTI | Ashanti |
| **brong-ahafo** | GHANA | Ghana | BONO | Brong-Ahafo |
| **brong-ahafo** | GHANA | Ghana | BONO EAST | Brong-Ahafo |
| **brong-ahafo** | GHANA | Ghana | BRONG AHAFO | Brong-Ahafo |
| **central** | GHANA | Ghana | CENTRAL | Central |
| **eastern** | GHANA | Ghana | EASTERN | Eastern |
| **greater accra** | GHANA | Ghana | GREATER ACCRA | Greater Accra |
| **northern** | GHANA | Ghana | NORTH EAST | ..Northern |
| **northern** | GHANA | Ghana | NORTHERN | ..Northern |
| **volta** | GHANA | Ghana | OTI | Volta |
| **northern** | GHANA | Ghana | SAVANNAH | ..Northern |
| **upper east** | GHANA | Ghana | UPPER EAST | ..Upper East |
| **upper west** | GHANA | Ghana | UPPER WEST | ..Upper West |
| **volta** | GHANA | Ghana | VOLTA | Volta |
| **western** | GHANA | Ghana | WESTERN | Western |
| **western** | GHANA | Ghana | WESTERN NORTH | Western |
| **boke** | GUINEA | Guinea | BOKE | Boké |
| **conakry** | GUINEA | Guinea | CONAKRY | Conakry |
| **faranah** | GUINEA | Guinea | FARANAH | Faranah |
| **kankan** | GUINEA | Guinea | KANKAN | Kankan |
| **kindia** | GUINEA | Guinea | KINDIA | Kindia |
| **labe** | GUINEA | Guinea | LABE | Labé |
| **mamou** | GUINEA | Guinea | MAMOU | Mamou |
| **nzerekore** | GUINEA | Guinea | NZEREKORE | N'Zérékoré |
| **coast** | KENYA | Kenya | COAST | Coast |
| **central** | KENYA | Kenya | KENCENTRAL | Central |
| **eastern** | KENYA | Kenya | KENEASTERN | Eastern |
| **western** | KENYA | Kenya | KENWESTERN | Western |
| **nairobi** | KENYA | Kenya | NAIROBI | Nairobi |
| **north eastern** | KENYA | Kenya | NORTH EASTERN | North Eastern |
| **nyanza** | KENYA | Kenya | NYANZA | Nyanza |
| **rift valley** | KENYA | Kenya | RIFT VALLEY | Rift Valley |
| **berea** | LESOTHO | Lesotho | BEREA | Berea |
| **butha-buthe** | LESOTHO | Lesotho | BUTHA-BUTHE | Butha-Buthe |
| **leribe** | LESOTHO | Lesotho | LERIBE | Leribe |
| **mafeteng** | LESOTHO | Lesotho | MAFETENG | Mafeteng |
| **maseru** | LESOTHO | Lesotho | MASERU | Maseru |
| **mohales hoek** | LESOTHO | Lesotho | MOHALES HOEK | Mohale's Hoek |
| **mokhotlong** | LESOTHO | Lesotho | MOKHOTLONG | Mokhotlong |
| **qachas nek** | LESOTHO | Lesotho | QACHAS NEK | Qasha's Nek |
| **quthing** | LESOTHO | Lesotho | QUTHING | Quthing |
| **thaba-tseka** | LESOTHO | Lesotho | THABA-TSEKA | Thaba-Tseka |
| **north western** | LIBERIA | Liberia | BOMI | North Western |
| **north central** | LIBERIA | Liberia | BONG | North Central |
| **north western** | LIBERIA | Liberia | GBARPOLU | North Western |
| **south central** | LIBERIA | Liberia | GRAND BASSA | South Central |
| **north western** | LIBERIA | Liberia | GRAND CAPE MOUNT | North Western |
| **south eastern a** | LIBERIA | Liberia | GRAND GEDEH | South Eastern A |
| **south eastern b** | LIBERIA | Liberia | GRAND KRU | South Eastern B |
| **north central** | LIBERIA | Liberia | LOFA | North Central |
| **south central** | LIBERIA | Liberia | MARGIBI | South Central |
| **south eastern b** | LIBERIA | Liberia | MARYLAND | South Eastern B |
| **south central** | LIBERIA | Liberia | MONTSERRADO | South Central |
| **north central** | LIBERIA | Liberia | NIMBA | North Central |
| **south eastern b** | LIBERIA | Liberia | RIVER GEE | South Eastern B |
| **south eastern a** | LIBERIA | Liberia | RIVERCESS | South Eastern A |
| **south eastern a** | LIBERIA | Liberia | SINOE | South Eastern A |
| **south central** | LIBERIA | Liberia |  | Monrovia |
| **central** | MALAWI | Malawi | CENTRAL | Central |
| **northern** | MALAWI | Malawi | NORTHERN | Northern |
| **southern** | MALAWI | Malawi | SOUTHERN | Southern |
| **cabo delgado** | MOZAMBIQUE | Mozambique | CABO DELGADO | Cabo Delgado |
| **maputo cidade** | MOZAMBIQUE | Mozambique | CIDADE DE MAPUTO | Maputo Cidade |
| **gaza** | MOZAMBIQUE | Mozambique | GAZA | Gaza |
| **inhambane** | MOZAMBIQUE | Mozambique | INHAMBANE | Inhambane |
| **manica** | MOZAMBIQUE | Mozambique | MANICA | Manica |
| **maputo cidade** | MOZAMBIQUE | Mozambique | MAPUTO CIDADE | Maputo Cidade |
| **maputo provincia** | MOZAMBIQUE | Mozambique | MAPUTO PROVINCIA | Maputo Provincia |
| **nampula** | MOZAMBIQUE | Mozambique | NAMPULA | Nampula |
| **niassa** | MOZAMBIQUE | Mozambique | NIASSA | Niassa |
| **sofala** | MOZAMBIQUE | Mozambique | SOFALA | Sofala |
| **tete** | MOZAMBIQUE | Mozambique | TETE | Tete |
| **zambezia** | MOZAMBIQUE | Mozambique | ZAMBEZIA | Zambézia |
| **abia** | NIGERIA | Nigeria | ABIA | ..Abia |
| **adamawa** | NIGERIA | Nigeria | ADAMAWA | ..Adamawa |
| **akwa ibom** | NIGERIA | Nigeria | AKWA IBOM | ..Akwa Ibom |
| **anambra** | NIGERIA | Nigeria | ANAMBRA | ..Anambra |
| **bauchi** | NIGERIA | Nigeria | BAUCHI | ..Bauchi |
| **bayelsa** | NIGERIA | Nigeria | BAYELSA | ..Bayelsa |
| **benue** | NIGERIA | Nigeria | BENUE | ..Benue |
| **borno** | NIGERIA | Nigeria | BORNO | ..Borno |
| **cross river** | NIGERIA | Nigeria | CROSS RIVER | ..Cross River |
| **delta** | NIGERIA | Nigeria | DELTA | ..Delta |
| **ebonyi** | NIGERIA | Nigeria | EBONYI | ..Ebonyi |
| **edo** | NIGERIA | Nigeria | EDO | ..Edo |
| **ekiti** | NIGERIA | Nigeria | EKITI | ..Ekiti |
| **enugu** | NIGERIA | Nigeria | ENUGU | ..Enugu |
| **FCT** | NIGERIA | Nigeria | FCT | ..FCT Abuja |
| **gombe** | NIGERIA | Nigeria | GOMBE | ..Gombe |
| **imo** | NIGERIA | Nigeria | IMO | ..Imo |
| **jigawa** | NIGERIA | Nigeria | JIGAWA | ..Jigawa |
| **kaduna** | NIGERIA | Nigeria | KADUNA | ..Kaduna |
| **kano** | NIGERIA | Nigeria | KANO | ..Kano |
| **katsina** | NIGERIA | Nigeria | KATSINA | ..Katsina |
| **kebbi** | NIGERIA | Nigeria | KEBBI | ..Kebbi |
| **kogi** | NIGERIA | Nigeria | KOGI | ..Kogi |
| **kwara** | NIGERIA | Nigeria | KWARA | ..Kwara |
| **lagos** | NIGERIA | Nigeria | LAGOS | ..Lagos |
| **nasarawa** | NIGERIA | Nigeria | NASARAWA | ..Nasarawa |
| **niger** | NIGERIA | Nigeria | NIGER | ..Niger |
| **ogun** | NIGERIA | Nigeria | OGUN | ..Ogun |
| **ondo** | NIGERIA | Nigeria | ONDO | ..Ondo |
| **osun** | NIGERIA | Nigeria | OSUN | ..Osun |
| **oyo** | NIGERIA | Nigeria | OYO | ..Oyo |
| **plateau** | NIGERIA | Nigeria | PLATEAU | ..Plateau |
| **rivers** | NIGERIA | Nigeria | RIVERS | ..Rivers |
| **sokoto** | NIGERIA | Nigeria | SOKOTO | ..Sokoto |
| **taraba** | NIGERIA | Nigeria | TARABA | ..Taraba |
| **yobe** | NIGERIA | Nigeria | YOBE | ..Yobe |
| **zamfara** | NIGERIA | Nigeria | ZAMFARA | ..Zamfara |
| **east** | RWANDA | Rwanda | EST | East |
| **kigali** | RWANDA | Rwanda | MVK | Kigali |
| **north** | RWANDA | Rwanda | NOR | North |
| **south** | RWANDA | Rwanda | OUE | South |
| **west** | RWANDA | Rwanda | SUD | West |
| **eastern** | SIERRA LEONE | Sierra Leone | EASTERN | Eastern |
| **northern and north western** | SIERRA LEONE | Sierra Leone | NORTH WESTERN | North Western |
| **northern and north western** | SIERRA LEONE | Sierra Leone | NORTHERN (21769-1) | Northern (before 2017) |
| **northern and north western** | SIERRA LEONE | Sierra Leone | NORTHERN (21769-1) | Northern |
| **southern** | SIERRA LEONE | Sierra Leone | SOUTHERN | Southern |
| **western** | SIERRA LEONE | Sierra Leone | WESTERN AREA | Western |
| **arusha** | UNITED REPUBLIC OF TANZANIA | Tanzania | ARUSHA | ..Arusha |
| **dar-es-salaam** | UNITED REPUBLIC OF TANZANIA | Tanzania | DAR ES SALAAM | ..Dar es Salaam |
| **dodoma** | UNITED REPUBLIC OF TANZANIA | Tanzania | DODOMA | ..Dodoma |
| **mwanza** | UNITED REPUBLIC OF TANZANIA | Tanzania | GEITA | ..Geita |
| **iringa** | UNITED REPUBLIC OF TANZANIA | Tanzania | IRINGA | ..Iringa |
| **kagera** | UNITED REPUBLIC OF TANZANIA | Tanzania | KAGERA | ..Kagera |
| **kaskazini pemba** | UNITED REPUBLIC OF TANZANIA | Tanzania | KASKAZINI PEMBA | ..Kaskazini Pemba |
| **kaskazini pemba** | UNITED REPUBLIC OF TANZANIA | Tanzania | KASKAZINI PEMBA | ..Pemba North |
| **kaskazini unguja** | UNITED REPUBLIC OF TANZANIA | Tanzania | KASKAZINI UNGUJA | ..Kaskazini Unguja |
| **kaskazini unguja** | UNITED REPUBLIC OF TANZANIA | Tanzania | KASKAZINI UNGUJA | ..Zanzibar North |
| **rukwa** | UNITED REPUBLIC OF TANZANIA | Tanzania | KATAVI | ..Katavi |
| **kigoma** | UNITED REPUBLIC OF TANZANIA | Tanzania | KIGOMA | ..Kigoma |
| **kilimanjaro** | UNITED REPUBLIC OF TANZANIA | Tanzania | KILIMANJARO | ..Kilimanjaro |
| **kusini pemba** | UNITED REPUBLIC OF TANZANIA | Tanzania | KUSINI PEMBA | ..Kusini Pemba |
| **kusini pemba** | UNITED REPUBLIC OF TANZANIA | Tanzania | KUSINI PEMBA | ..Pemba South |
| **kusini unguja** | UNITED REPUBLIC OF TANZANIA | Tanzania | KUSINI UNGUJA | ..Kusini Unguja |
| **kusini unguja** | UNITED REPUBLIC OF TANZANIA | Tanzania | KUSINI UNGUJA | ..Zanzibar South |
| **lindi** | UNITED REPUBLIC OF TANZANIA | Tanzania | LINDI | ..Lindi |
| **manyara** | UNITED REPUBLIC OF TANZANIA | Tanzania | MANYARA | ..Manyara |
| **mara** | UNITED REPUBLIC OF TANZANIA | Tanzania | MARA | ..Mara |
| **mbeya** | UNITED REPUBLIC OF TANZANIA | Tanzania | MBEYA (21769-2) | ..Mbeya |
| **mbeya** | UNITED REPUBLIC OF TANZANIA | Tanzania | MBEYA (21769-2) | ..Mbeya (before 2016) |
| **mjini magharibi** | UNITED REPUBLIC OF TANZANIA | Tanzania | MJINI MAGHARIBI | ..Mjini Magharibi |
| **mjini magharibi** | UNITED REPUBLIC OF TANZANIA | Tanzania | MJINI MAGHARIBI | ..Town West |
| **morogoro** | UNITED REPUBLIC OF TANZANIA | Tanzania | MOROGORO | ..Morogoro |
| **mtwara** | UNITED REPUBLIC OF TANZANIA | Tanzania | MTWARA | ..Mtwara |
| **mwanza** | UNITED REPUBLIC OF TANZANIA | Tanzania | MWANZA | ..Mwanza |
| **iringa** | UNITED REPUBLIC OF TANZANIA | Tanzania | NJOMBE | ..Njombe |
| **pwani** | UNITED REPUBLIC OF TANZANIA | Tanzania | PWANI | ..Pwani |
| **rukwa** | UNITED REPUBLIC OF TANZANIA | Tanzania | RUKWA | ..Rukwa |
| **ruvuma** | UNITED REPUBLIC OF TANZANIA | Tanzania | RUVUMA | ..Ruvuma |
| **shinyanga** | UNITED REPUBLIC OF TANZANIA | Tanzania | SHINYANGA | ..Shinyanga |
| **shinyanga** | UNITED REPUBLIC OF TANZANIA | Tanzania | SIMIYU | ..Simiyu |
| **singida** | UNITED REPUBLIC OF TANZANIA | Tanzania | SINGIDA | ..Singida |
| **mbeya** | UNITED REPUBLIC OF TANZANIA | Tanzania | SONGWE |  |
| **tabora** | UNITED REPUBLIC OF TANZANIA | Tanzania | TABORA | ..Tabora |
| **tanga** | UNITED REPUBLIC OF TANZANIA | Tanzania | TANGA | ..Tanga |
| **central** | ZAMBIA | Zambia | CENTRAL (21769-1) | Central |
| **copperbelt** | ZAMBIA | Zambia | COPPERBELT (21769-1) | Copperbelt |
| **northern and eastern and muchinga** | ZAMBIA | Zambia | EASTERN | Eastern |
| **luapula** | ZAMBIA | Zambia | LUAPULA (21769-1) | Luapula |
| **lusaka** | ZAMBIA | Zambia | LUSAKA (21769-3) | Lusaka |
| **northern and eastern and muchinga** | ZAMBIA | Zambia | MUCHINGA | Eastern (before 2011) |
| **north-western** | ZAMBIA | Zambia | NORTH WESTERN | North-Western |
| **north-western** | ZAMBIA | Zambia | NORTH-WESTERN |  |
| **northern and eastern and muchinga** | ZAMBIA | Zambia | NORTHERN | Northern |
| **southern** | ZAMBIA | Zambia | SOUTHERN | Southern |
| **western** | ZAMBIA | Zambia | WESTERN | Western |
| **central** | ZAMBIA | Zambia | ZAMCENTRAL |  |
| **northern and eastern and muchinga** | ZAMBIA | Zambia | ZAMEASTERN | Northern (before 2011) |
| **northern and eastern and muchinga** | ZAMBIA | Zambia | ZAMNORTHERN |  |
| **southern** | ZAMBIA | Zambia | ZAMSOUTHERN |  |
| **western** | ZAMBIA | Zambia | ZAMWESTERN |  |
| **northern and eastern and muchinga** | ZAMBIA | Zambia |  | Muchinga |
| **bulawayo** | ZIMBABWE | Zimbabwe | BULAWAYO | Bulawayo |
| **harare** | ZIMBABWE | Zimbabwe | HARARE | Harare Chitungwiza |
| **manicaland** | ZIMBABWE | Zimbabwe | MANICALAND | Manicaland |
| **mashonaland central** | ZIMBABWE | Zimbabwe | MASHONALAND CENTRAL | Mashonaland Central |
| **mashonaland east** | ZIMBABWE | Zimbabwe | MASHONALAND EAST | Mashonaland East |
| **mashonaland west** | ZIMBABWE | Zimbabwe | MASHONALAND WEST | Mashonaland West |
| **masvingo** | ZIMBABWE | Zimbabwe | MASVINGO | Masvingo |
| **matabeleland north** | ZIMBABWE | Zimbabwe | MATABELELAND NORTH | Matabeleland North |
| **matabeleland south** | ZIMBABWE | Zimbabwe | MATABELELAND SOUTH | Matabeleland South |
| **midlands** | ZIMBABWE | Zimbabwe | MIDLANDS | Midlands |

Supplementary Table 5: Aggregations of study area ADM1 regions, reconciling DHS and WHO datasets.

|  | Coefficient | | | |
| --- | --- | --- | --- | --- |
| *Estimate type* | Intercept | Proportion previously vaccinated | Mean age | Proportion testing IgM negative |
| *Estimate using mean DHS coverage value* | -0.3796 | 1.6355 | 0.0855 | 0.0486 |
| *p value of estimate using mean DHS coverage value* | 0.1703 | 0.0000 | 0.0013 | 0.8830 |
| *Bootstrap mean estimate value* | -0.3664 | 1.6056 | 0.0835 | 0.0311 |
| *2.5th percentile of bootstrap estimates* | -0.5423 | 1.3740 | 0.0636 | -0.2125 |
| *97.5th percentile of bootstrap estimates* | -0.1694 | 1.7832 | 0.1025 | 0.2748 |

Supplementary Table 6: Parameter estimates for the beta regression models. The first two rows describe estimates derived from a single model, using the DHS-derived coverage values for the first DHS survey iteration for each country. The third through fifth rows describe the distribution of estimates derived from 100 model runs that sampled from a distribution of outcome values for each run.
